# Unexpected Infective Endocarditis: Towards a New Alert for Clinicians

**DOI:** 10.3390/jcm13175058

**Published:** 2024-08-26

**Authors:** Giovanni La Canna, Lucia Torracca, Alessandro Barbone, Iside Scarfò

**Affiliations:** 1 Applied Diagnostic Echocardiography, IRCCS Humanitas Clinical and Research Hospital, 20089 Rozzano, Milan, Italy; iside.scarfo@humanitas.it; 2 Cardiac Surgery Department, IRCCS Humanitas Clinical and Research Hospital, 20089 Rozzano, Milan, Italy; lucia.torracca@humanitas.it (L.T.); alessandro.barbone@humanitas.it (A.B.)

**Keywords:** infective endocarditis, emergent at-risk population, subtle clinical phenotypes, unexpected diagnostic challenges

## Abstract

Despite the clear indications and worldwide application of specific guidelines, the recognition of Infective Endocarditis (IE) may be challenging in day-to-day clinical practice. Significant changes in the epidemiological and clinical profile of IE have been observed, including variations in the populations at risk and an increased incidence in subjects without at-risk cardiac disease. Emergent at-risk populations for IE particularly include immunocompromised patients with a comorbidity burden (e.g., cancer, diabetes, dialysis), requiring long-term central venous catheters or recurrent healthcare interventions. In addition, healthy subjects, such as skin-contact athletes or those with piercing implants, may be exposed to the transmission of highly virulent bacteria (through the skin or mucous), determining endothelial lesions and subsequent IE, despite the absence of pre-existing at-risk cardiac disease. Emergent at-risk populations and clinical presentation changes may subvert the conventional paradigm of IE toward an unexpected clinical scenario. Owing to its unusual clinical context, IE might be overlooked, resulting in a challenging diagnosis and delayed treatment. This review, supported by a series of clinical cases, analyzed the subtle and deceptive phenotypes subtending the complex syndrome of unexpected IE. The awareness of an unexpected clinical course should alert clinicians to also consider IE diagnosis in patients with atypical features, enhancing vigilance for preventive measures in an emergent at-risk population untargeted by conventional workflows.

## 1. Introduction

Infective Endocarditis (IE) is an uncommon disease affecting 3–10 per 100,000 people per year [1]. Although there has been specific progress in diagnosis and therapeutic strategies, IE is burdened by a high overall 1-year mortality rate (almost 30%) [2,3,4]. Prompt diagnosis, together with timely and appropriate treatment, are key factors in improving adverse IE prognosis. Standard diagnosis is based on pathological criteria, including histology or culture from material obtained at autopsy or during cardiac surgery. In the absence of standard pathological findings, in clinical practice, definite or probable IE diagnosis is based on varying criteria, as suggested in the specific guidelines [5,6]. Despite the clear indications and worldwide application of these guidelines, IE recognition may be challenging in day-to-day clinical practice. Varying predictive scores have been proposed to define IE clinical expectations and guide the subsequent imaging workflow [7,8]. However, in recent years, IE has progressively changed face [9,10,11,12,13,14,15], challenging conventional preventive and diagnostic work-up based on expected disease probability. Indeed, significant changes in the epidemiological and clinical profile of IE have been observed, including variations in the population at risk and increased incidence in subjects without at-risk cardiac diseases. Thus, emerging at-risk groups (especially patients with comorbidity burdens) are untargeted by risk-score models, leading to potentially misleading work-up. For example, imaging techniques focusing on endocardial valve damage may be omitted due to an unexpected IE clinical context. Unexpected IE has been described as a surprising finding in clinical case reports [16,17,18], but comprehensive analyses of clinical settings, which should alert for IE beyond the conventional recognized paradigm, have not yet been published. Therefore, this review, supported by a focused clinical case series, aims to highlight the unexpected IE scenario, attempting to refine a subset of patients requiring preventive and diagnostic pathways that could otherwise be unattended or delayed. We performed a literature search of the PubMed database using the search terms “endocarditis” or “infective endocarditis”, in conjunction with “epidemiology”, “pathogenesis”, “comorbidity”, “clinical manifestations”, “imaging”, “diagnosis”. Articles deemed to be relevant were selected and included in our review. Only articles published in English were considered.

## 2. Infective Endocarditis Pathophysiology

The initial step of IE is bacteremia, a consequence of bacteria entry into the bloodstream via the skin through direct contact, venous catheter insertion, or following invasive procedures, or via the mouth, or else through the gastrointestinal and urinary tracts. After entering the bloodstream, bacteria are recognized by endothelial receptors, leukocytes, and platelets, with cytokine release and subsequent prothrombotic–phlogistic state induction [19,20,21,22,23]. Activated endothelial cells and leukocytes release tissue factors, which in turn trigger the extrinsic coagulation pathway. In addition, the intrinsic coagulation pathway is also activated due to stimulated factor XII caused by dying cells or bacterial cell-wall components, which in turn activates the proinflammatory bradykinin-producing kallikrein system and binds coagulation with the complement system. The activated coagulation cascade promotes prothrombin–thrombin cleaving, which activates platelets and fibrin. Subsequent fibrin-related infective tissue sealing-off limits further bacterial spreading. Platelets kill bacteria with alpha-granule-derived platelet microbicidal proteins, further fueling coagulation, stimulating neutrophil extracellular trap formation and, together with leukocytes, further coordinating the immune response by secreting various cytokines. Despite being triggered in an attempt to limit the invading infection, the immunological and thrombosis process may also become an optimal environment for bacterial survival and growth [20]. According to the accepted pathogenetic model, IE development requires a pre-existing mechanical or inflammatory endothelial lesion, favoring microbial adherence to the injured endothelium during bacteremia. Bacteria adherence capability is modulated by specialized proteins (adhesins) and facilitated by fibrin and platelet microthrombi [21]. Pathogens that carry fibronectin-binding proteins on their surface enhance the bacterial adherence capability, promoting a mechanism of IE development also in patients without pre-existing cardiac valve disease [23]. *Staphylococcus aureus* endothelial infection following bacteremia has been analyzed in experimental models. *S. aureus* can produce a surprising number of mechanisms against immune host defenses. Owing to its complex and effective immuno-evasion of host defenses, *S.aureus* can induce primary endothelial damage even in the absence of a pre-existing lesion. Endothelial disruption contact of blood with subendothelial factors (extracellular matrix proteins, thromboplastin, and tissue factors) promotes coagulation, and pathogens bind to the resultant coagulum. Subsequently, a cycle of monocyte activation and cytokine and tissue factor production may lead to progressive enlargement of infected vegetation, tissue damage, and abscess formation. In addition, clumps of infected vegetation may break away from the primary infection site and emboli may disseminate to remote organs, notably the brain, spleen, and kidney, with corresponding resultant clinical sequelae.

## 3. The Diagnosis of Infective Endocarditis: The Expected Paradigm

According to specific AHA/ACC and ESC Guidelines [5,6], the groups of subjects at high risk of IE can be defined based on the following criteria: (1) Patients with previous IE. (2) Patients with surgically or transcatheter implanted prosthetic valves, and with any material used for cardiac valve repair (septal defect closure devices, left atrial appendage closure devices, vascular grafts, vena cava filters, and central venous system ventriculoatrial shunts are considered within this risk category during the first 6 months after implantation). (3) Patients with congenital heart disease (CHD) (not including isolated congenital valve abnormalities). The risk is high in untreated cyanotic CHD or in groups surgically treated with prosthetic material, including valved conduits or systemic to pulmonary shunts (CHD patients undergoing transcatheter correction of an atrial or ventricular septal defect using devices or surgery with non-valve-related prosthetic materials are at risk for the subsequent first 6 months). (4) patients with ventricular assist devices, such as destination therapy, are considered at high risk. These patients should undergo antibiotic prophylaxis at the time of procedures which are at risk of bacteremia.

Patients at intermediate risk of IE include (1) rheumatic heart disease or nonrheumatic degenerative valve disease; (2) congenital valve abnormalities, including bicuspid aortic valve disease; (3) cardiovascular implanted electronic devices (CIEDs); and (4) hypertrophic cardiomyopathy. In patients at intermediate risk of IE, prevention measures are strongly encouraged, but antibiotic prophylaxis is not routinely recommended.

Pre-existing cardiac conditions at high or intermediate risk can also support an estimation of disease probability to guide IE diagnostic pathways in an expected clinical context [15]. Standard diagnosis of IE is based on pathological criteria, including histology or culture from material obtained at autopsy or cardiac surgery. In the absence of standard pathological findings, IE diagnosis is based on clinical Duke criteria as suggested in specific guidelines, including major criteria (positive blood culture for recognized typical microorganisms and imaging evidence of endothelial valve involvement in the absence of an alternative explanation) and minor criteria, such as predisposing heart disease, fever >38°, immunological and vascular phenomena, embolic vascular dissemination (including those asymptomatic, detected by imaging only), immunological phenomena (Glomerulonephritis, Osler nodes and Roth spots, Rheumatoid factor), microbiological evidence (positive blood culture but does not meet a major criterion as noted above, serological evidence of active infection with organism consistent with IE). The consistency between clinical suspicion and the two major criteria (typical bacteremia and diagnostic imaging) may lead to a definite diagnosis of IE. Definite IE diagnosis may also be supported by at least three minor criteria in the absence of one of the two major criteria, or by five minor criteria alone. Possible IE diagnosis requires a combination of one major criterion and at least one minor criterion or three minor criteria alone. In the absence of a criteria combination indicating a definite or possible diagnosis, IE is rejected. Duke criteria revision was proposed at the 2023 Duke International Society for Cardiovascular Infectious Diseases Diagnostic Criteria for Infective Endocarditis (Duke-ISCVID) [24], including new microbiology diagnostics (enzyme immunoassay for Bartonella and Brucella species, polymerase chain reaction, amplicon/metagenomic sequencing, in situ hybridization), imaging (positron emission computed tomography with 18F-fluorodeoxyglucose, cardiac computed tomography), and the inclusion of intraoperative inspection as a new major clinical criterion. In addition, DUKE-ISCVID expanded the list of the “typical” microorganisms causing IE only in the presence of intracardiac prostheses. Finally, transcatheter valve implants and endovascular cardiac implantable electronic devices have been added to cardiac predisposing criteria, and cerebral or splenic abscess to vascular phenomena. Two recent large clinical studies have reported an improvement in the diagnostic accuracy of Duke-ISCVID in comparison with the 2000 modified Duke criteria and the 2015 ESC criteria. However, the Amsterdam data reported by van der Vaart TW et al. [25] show suboptimal sensitivity (75%) of surgically confirmed IE supporting a high percentage of patients who are not correctly labeled. Probably, the omission of TEE in a significant group (25%) could have lowered the sensitivity for IE diagnosis. On the other hand, the Switzerland data report an increase in sensitivity (from 70% to 84%) together with a decreasing specificity (from 74% to 60%) [26]. The appropriate use and modality of cardiac imaging (transthoracic vs. transesophageal echocardiography, nuclear medicine techniques, computed tomography) are crucial for the detection of endocarditis-related valve lesions [27]. In addition to guideline suggestions, several risk score models have been proposed to refine predisposed clinical conditions for an effective IE diagnostic workflow. In particular, some scoring models have been tested in clinical trials to assess TEE cost-effectiveness in subjects with *S. aureus* [28,29,30] or enterococcal bacteremia [31,32,33,34] (Table S1 Supplement). Despite heterogeneous variables, proposed scoring systems provide high negative predictive values but low specificity in defining an IE clinical expectation threshold for the subsequent TEE. Low disease prevalence in the tested population could have affected the post-test outcome of the scoring models. Obviously, changing the scoring variables may improve the sensitivity or specificity of predictive models, avoiding underdiagnosis or overdiagnosis, respectively. Primarily, retrospective study analysis and panel consensus, rather than pathologically confirmed IE as an endpoint together with tertiary hospital settings, limit the inference of published data in the general population and in all clinical settings. Thus, the workflow for IE should consider the population that could not be captured by the proposed clinical scoring. Clinical diagnosis of IE requires the combination of several variables, including the presence of an at-risk cardiac condition, together with multimodality imaging and microbiological criteria consistency. The expected paradigm is based on a typical clinical presentation triggering a focused and skillful diagnostic work-up in a high-risk context. However, in the context of clinical practice, this paradigm may be influenced by several confounding conditions, which can reduce the accuracy of conventional pathways for prompt IE diagnosis and treatment. Notably, a clinical threshold triggering cardiac imaging indications, hospital resource availability, and competence in the interpretation of imaging findings may impact guideline criteria accuracy in IE diagnosis. Even though it is one of the two major criteria for IE diagnosis, bacteremia requires an integrated clinical evaluation of concomitant extracardiac infective foci or factors determining negative blood culture (previous antibiotic therapy, fastidious microbes, or fungi).

## 4. The Diagnosis of Infective Endocarditis: The Unexpected Scenario

Specific guidelines have substantiated effective pathways for IE diagnosis in expected clinical contexts [5,6]. However, in recent years, IE has progressively changed face [9,10,11,12,13,14,15]. Variations in at-risk populations and an increased incidence in subjects without at-risk cardiac disease particularly challenge the conventional diagnostic work-up of IE. Based on published data, up to 50% of IE occurs in subjects without underlying cardiac conditions (UCC) [35,36,37,38]. Apart from the expected probability based on high- or intermediate-risk criteria targeting conventional prophylaxis strategies, guideline parameters may not capture a large population at risk of IE development. According to a recent study, IE patients without UCC show a higher incidence of noncardiac comorbidities (e.g., immune deficiency, cancer) or a need for long-term central venous catheters, and different causative microorganisms in comparison with those with UCC [35]. Notably, patients without UCC may have large vegetations with related embolic complications due to delayed diagnosis and therapy. Paradoxically, IE patients without pre-existent UCC may be exposed to a greater risk of complications than those with UCC owing to misleading work-up in an unexpected clinical context. Well-designed epidemiological studies on IE incidence in new at-risk populations are scarce. Observational retrospective Spanish data report an increased incidence of IE patients between 2001 and 2014 (compared with 1987 to 2001) from 2.7 to 3.49 per 100,000 subjects per year, the rise being higher among older adults with comorbidities; patients without pre-existing heart disease were normally older adults with immunosuppression who were infected after exposure to healthcare settings [14,36]. A population-based observational study in France reported an annual incidence of IE of 33.8 cases per million [39]; the highest incidence was noted in men between 75 and 79 years old in the absence of known prior heart disease, with healthcare-associated IE accounting for 27% of cases. A prospective international observational study reported *S. aureus* to be the most common pathogen among the enrolled 1779 cases of definite IE, with a large regional variation in the at-risk population. Notably, hemodialysis, diabetes, and intravascular devices were the most common factors associated with IE in the United States compared with other countries [40]. The incidence of predisposing conditions, such as rheumatic heart disease or injected drug use, an older adult population, and healthcare systems, might vary over time and among regions, depending also on whether it is in low- or high-income countries.

### 4.1. Infective Endocarditis in Healthy Subjects

In healthy people, cardiac valve endothelium is highly resistant to infection. However, endothelial inflammation, even in the absence of pre-existent mechanical damage, renders cardiac valves vulnerable to infection following the exposure of healthy subjects to highly virulent agents [23]. When endothelial inflammation, rather than a pre-existent mechanical lesion, is the main predisposing risk condition, *S. aureus* is the predominant pathogen of IE.

#### 4.1.1. Athletes

Athletes are exposed to infections, particularly *S. aureus*, mainly due to skin-lesion-related entry points during physical contact sports [41,42,43,44]. In addition to skin wounds, space sharing and lack of hygiene are important factors favoring staphylococcal infection and transmission [45]. A significant methicilin-resistant *S. aureus* (MRSA) infection was found in asymptomatic athletes, with up to a 13% prevalence in college athletes. The prevalence of MRSA among college athletes was twice that found in patients in an intensive care unit and similar to that found in dialysis patients or patients with HIV [42]. Colonization of MRSA serves as a reservoir for transmission among athletes and can occur not only through contact with the contaminated wounds of infected patients but also via exposure to droplets from nasal carriers, colonized intact skin, or contaminated objects. Athletes with MRSA may be exposed to recurrent bacteriemia and subsequent endothelium phlogosis, triggering susceptibility to valve infection and related complications. It is mandatory to focus on preventive strategies, including eradication of MRSA in the entire team, good personal hygiene, cleaning and dressing of wounds, and avoiding the sharing of objects that come into contact with the skin. Notably, patients with asymptomatic nonsevere cardiac disease are not denied sports activities. Even though preventive antibiotic prophylaxis is not recommended, healthy subjects or patients with moderate cardiac disease may be exposed to highly virulent infection and bacteriemia during sports with infected skin lesion contact, leading to potentially aggressive bacteria adhesion to the endothelium and subsequent risk of IE development [41,42,43,44,45].

#### 4.1.2. Piercing

Body piercing is largely carried out in the general population, with common sites being the ears, mouth, nose, eyebrows, nipples, navel, and genitals [46,47]. Body piercing may be complicated by localized infective cellulitis sustained by skin bacterial flora, including the staphylococcal and streptococcal species. Cartilaginous ear and nasal structure piercing implantations are associated with a high incidence of pseudomonas infections, while genital piercings are at increased risk for sexually transmitted infections, such as *Neisseria gonorrhea* and *Chlamydia trachomatis*. Additionally, patients colonized with *S. aureus* are at increased risk of nasal piercing infections. Of those individuals with piercings at sites other than the soft earlobe, 23% reported a medical complication, especially when piercing was performed outside of a certificated body art studio. Skin and soft tissue complications will present similarly to localized cellulitis infections or abscesses. When disseminated infection occurs, systemic symptoms such as fever, tachycardia, and malaise may be present. Complicated piercings may become an entry site for bacteria (especially *S. aureus*) in the bloodstream, and endocarditis may occur due to the possible dissemination to distant sites. Intravenous drug abusers are exposed to a higher risk of IE related to piercing infections [46,47,48,49]. See Clinical Case 1.

Clinical Case 1. A 38-year-old woman without pre-existing cardiac disease was admitted for 10-day mild fever associated with progressive epigastric pain and vomit one month following piercing implant. Clinical examination revealed a painful hepato-splenomegaly. Cardiovascular evaluation showed tachycardia (120 beat/min), arterial hypotension (75/45 mm Hg) without any cardiac murmur. Abdomen echo scan confirmed a normally structured hepatosplenomegaly, together with inferior vena cava enlargement suggesting venous congestion. Blood chemistry showed increased white cell count (12,860) and severe anemia (Hb3.9), requiring urgent transfusion. C-reactive protein was moderately high (16.5). Blood culture was positive for methicillin sensitive *S. aureus*. TT (**1**) and subsequent TEE (**2**,**3**,**4**) showed a large iso-echogenic vegetation (yellow arrow), which was attached to the atrial surface of the anterior leaflet of the tricuspid valve, prolapsing into the right atrium during the systole and determining related severe valve regurgitation. The patient underwent culture-guided antibiotic therapy with disappearance of the vegetation and reduction in tricuspid regurgitation to a moderate degree at subsequent echocardiographic examination.

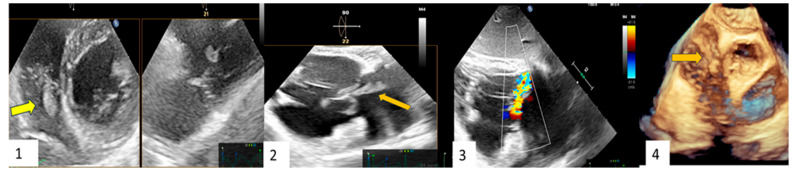


### 4.2. Comorbidities and Infective Endocarditis

Comorbidities are relatively common in IE patients [50]. Notably, comorbidities may be a dominant pre-existing clinical feature and can significantly impact the diagnostic work-up, course, and outcome of IE.

#### 4.2.1. Diabetes

Even though not targeted by conventional preventive strategies, diabetic patients are at high risk of developing IE compared with the general population [51,52,53,54,55]. Diabetic patients have an increased susceptibility to infections, including a higher rate of skin and soft tissue infections, urinary tract infections, respiratory tract infections, and periodontal disease, all of which can be a source of bacteremia. Patients who are closely followed in out-patient clinics and undergo healthcare interventions with invasive procedures are exposed to higher IE risk [50,55]. Indeed, an increase in staphylococcal etiology and higher nosocomial or healthcare-associated IE has been reported in diabetic patients. Furthermore, diabetes-associated severe endothelial dysfunction and an impaired immune response play a central pathophysiologic role in the development of IE, favoring bacteria adhesion and vegetation growth, as well as in the absence of pre-existing valve pathologies [51,52,53,54,55,56,57,58,59,60,61,62]. The duration and late-stage complications of diabetes increase the risk of IE [58]. Endothelial dysfunction is related to oxidative stress, with an increase in harmful free radicals, chronic inflammation, and reduced nitric oxide availability. The immune capacity may be impaired due to several reasons, including (1) high blood glucose levels, which can impair the function of white blood cells; (2) reduced blood flow as a consequence of vascular damage, which reduces the delivery of immune cells to infection sites, delaying the healing process; (3) nerve damage, which can reduce sensation, delaying infection treatment; (4) chronic inflammation; (5) altered immune cell function; and (6) compromised immune barriers. Due to a commonly altered immune response in diabetic patients, the clinical presentation of IE can sometimes be atypical. Fever response may be blunted and nonspecific symptoms, such as fatigue or weight loss, might be mistakenly attributed to diabetes itself rather than to infection, making IE diagnosis more challenging. In addition, diabetic patients might experience more right-sided infections, especially if they have a history of intravenous drug use. Preventive measures, such as good glycemic control, good dental hygiene, careful monitoring for infective foci, as well as systematic hygiene surveillance during access to healthcare facilities and hospitalization are paramount in diabetic patients.

#### 4.2.2. Hemodialysis Patients

Chronic hemodialysis has emerged as a condition at risk for IE development, even in the absence of predisposing cardiac disease [63]. Susceptibility to IE of patients undergoing hemodialysis is related to several factors, including clinical patient profile (immuno-incompetence, comorbidities, malnutrition, frailty); exposure to recurrent microbiological contamination, arising from a repeated need for vascular access, indwelling catheters, native AV fistula or graft (which can be a portal for bacterial entry); and highly virulent community, healthcare, or nosocomial infections with a high incidence of methicillin-resistant *S. aureus* [63,64,65,66,67,68]. According to the International Collaboration on Endocarditis Database [67], the incidence of IE is 8% and is related to healthcare-associated or nosocomial infection with Staphylococci (47.8%) and Enterococci (15.4%). Indwelling catheters, together with AV native fistulas or AV grafts, are the most important factors associated with IE development. Bloodstream bacteria entry may result from a patient’s own cutaneous flora (in particular staphylococcal infections) or from exogenous sources (e.g., the hands of personnel, contaminated equipment). The clinical presentation of IE in the hemodialysis population may be subtle with low-grade or absent fever, moderate dyspnea, and weakness. In the advanced clinical stage, patients may present congestive heart failure, requiring an increase in load subtraction during dialysis resulting from valve dysfunction overload, or exhibit signs of acute extracardiac embolism or subtle back pain due to the development of spondylodiscitis. Due to subtle clinical pictures, suspicion of IE may be overlooked, especially in the presence of apparently uncomplicated indwelling catheter infection. Notably, the diagnosis of IE in hemodialysis patients using bacteremia as the major Duke criteria for IE is challenged by the presence of access catheter contamination as a removable source of infection, precluding the diagnostic power of bacteremia. Due to the low grade or absence of fever (50%) and uncaptured risk condition from the conventional score, even in the presence of bacteremia advanced diagnostic imaging may be omitted, resulting in delayed IE diagnosis. Thus, conclusive IE diagnosis in hemodialysis patients may be unexpected, occurring in an untargeted clinical context. Preventive strategies are crucial, including meticulous care of vascular access, close adherence to infection control measures, and prompt treatment of infective foci.

#### 4.2.3. Cancer Patients

Several factors favor IE development in cancer patients without underlying at-risk cardiac disease. Immuno-incompetence (as a consequence of cancer itself or the side effects of chemotherapy) and hypercoagulability (mainly due to tumor-released procoagulant substances in the setting of paraneoplastic syndrome and the effects of cancer treatment) may lead to increased vulnerability to infection. Intravascular devices, which are needed for chemotherapy or nutritional support, may be colonized by bacteria, providing a direct pathway for pathogens to enter the bloodstream and potentially infect the heart valve. Additional factors include mucosal damage due to chemotherapy and radiotherapy favoring bacterial entry into the bloodstream, frequent hospitalization, and recurrent local or systemic infections [69,70,71,72]. According to published data in a retrospective study, up to 18% of IE prevalence was reported in cancer patients with different causative organisms (a higher incidence of staphylococcal and enterococcal infections than in noncancer patients) [71]. Recently, the multicentric prospective ESC EORP European Endocarditis (EURO-ENDO) registry reported a high prevalence (11.6%) of cancer in IE patients with causative organisms, including *S. aureus* and Enterococci, which were mainly community-acquired and preceded by nondental procedures [70]. In cancer patients, IE is a complex clinical issue due to a compromised health status and clinical overlapping with symptoms related to cancer and its specific treatment. Notably, symptoms such as fever, fatigue, or weight loss could be attributed to the cancer and its treatment, potentially delaying IE diagnosis. Hypercoagulability may lead to the development of nonbacterial thrombotic endocarditis (NBTE) (also called marantic endocarditis) [73,74,75,76,77,78], which is characterized by the deposition of sterile thrombi on cardiac valves. However, NBTE becomes a site for secondary bacterial colonization leading to infective endocarditis. A diagnosis of IE may be challenging due to coexistent thrombosis and an intriguing or atypical clinical presentation. In addition, a high incidence of blood culture-negative endocarditis (up to 42%) further enhances the complexity of distinguishing true culture-negative IE from marantic endocarditis, requiring a high degree of clinical suspicion, with definitive diagnosis often made during surgery or autopsy [72,79,80].

### 4.3. Subclinical Valve Disease in the Elderly

The elderly are exposed to natural heart valve degeneration, which can create an environment susceptible to IE also in the absence of clinically significant hemodynamic damage. Subclinical valve disease is commonly undetectable with routine clinical examinations but may be recognized by echocardiography [81]. These subtle abnormalities can favor bacterial adherence and IE development, especially by highly virulent nosocomial infections, poor dental health, urinary tract infections, and invasive procedures. Clinical pictures in the elderly are subtle, such as malaise, fever, and anorexia overlapping other common conditions, such as viral infections and frailty. Subclinical valve disease in elderly subjects is associated with a risk of IE development, although this risk is often underestimated. Increased awareness and vigilance for symptoms suggestive of IE are important in this population, especially considering potentially atypical presentations and challenges regarding diagnosis and management [82,83,84,85,86,87,88,89].

## 5. Unexpected Infective Endocarditis Syndrome: An Intriguing Clinical Challenge

**Figure 1:**
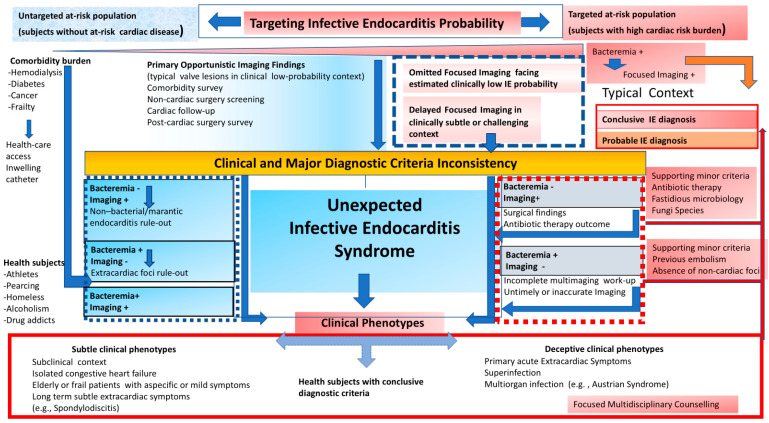
Perspectives on Infective Endocarditis diagnosis facing expected (red work-flow) and unexpected (blue work-flow) clinical scenarios. Dashed-line boxes include clinical and major diagnostic criteria inconsistency subtending unexpected Infective Endocarditis syndrome with respective multiple phenotypes.

Several confounding factors may impact the accuracy of the conventional criteria for IE in day-to-day clinical practice, resulting in a growing percentage of patients with unexpected diagnoses. Clinical and diagnostic criteria mismatch may challenge the typical paradigm of IE toward a varying scenario of unexpected IE syndrome. According to Figure 1, diagnostic IE workflow is commonly engaged by the clinical suspicion of disease. A typical scenario involves patients with a high probability of clinical disease due to underlying at-risk heart disease, bacteremia, and typical symptoms needing diagnostic tests to confirm infective valve involvement. Varying scores are used to establish the probability threshold of disease to guide subsequent IE diagnostic pathways [7,8,15,28,29,30,31,32,33,34]. The most well-known scoring system to determine the likelihood of endocarditis is the Modified Duke Criteria, based on clinical, microbiological, and imaging findings [5,6]. The characteristics used to define IE risk include pre-existent at-risk cardiac disease, intracardiac devices, prosthetic valves, and congenital heart disease. However, ultimate IE diagnosis is mainly affected by the completeness and time of focused imaging work-up. Based on the clinical probability of IE, together with typical bacteremia (i.e., *S. aureus*), a patient can undergo subsequent cardiac imaging to detect endocardial involvement. Transthoracic echocardiography is the first-line imaging modality supplemented with an additional transesophageal approach to corroborate diagnosis and guide IE management. Depending on the clinical context, for example, prosthetic valve or vascular graft, the patient may require further examination with a CT or PET scan. Simultaneous “in parallel” multi-imaging may be cost-ineffective. The consistency between clinical suspicion and the two major criteria (typical bacteremia and diagnostic imaging) may lead to a conclusive diagnosis of IE. In the context of high clinical probability, the management of inconsistency between two major criteria may be challenging. Under antibiotic therapy, or due to fastidious microbes or fungi species, blood culture may be negative, despite diagnostic imaging suggesting IE. Minor Duke criteria may support IE diagnosis in the context of a mismatch between suggestive imaging of valve involvement and negative blood culture. Pathological findings at the time of surgery, when indicated, and a favorable trajectory of valve vegetation under antibiotic therapy can lead to a definite IE diagnosis. Thus, in the presence of typical valve involvement and related complications, negative blood culture cannot reject IE. Systematic serological testing for *Coxiella Burnetii*, *Bartonella* spp., *Aspergillus* spp., *Mycoplasma pneumoniae*, *Brucella* spp. and *Legionella pneumophila* should be proposed, followed by specific polymerase chain reaction (PCR) assays for *Tropheryma whipplei*, *Bartonella* spp. and fungi (*Candida* spp., *Aspergillus* spp.) from blood and tissue [6]. Furthermore, as reported in the workflow, in the arm of high clinical IE suspicion, negative cardiac imaging may be related to inappropriately early examination, poor imaging quality, previous embolism, or incomplete multi-imaging examinations. Repeated negative transesophageal echocardiography and alternative imaging, despite persistent bacteremia and clinical suspicion, do not reject IE diagnosis. Particularly, in the context of high-risk patients, cardiac imaging may be omitted or delayed due to an estimated low probability of IE owing to subtle or challenging clinical features, leading to inconsistency between imaging and microbiological criteria. Valve damage or uncontrolled sepsis may require surgery, which could confirm unexpected IE.

An important percentage of IE may occur despite the absence of conventional high- or intermediate-risk cardiac diseases, resulting in an unexpected finding [35,36]. This population group may particularly include patients with a comorbidity burden (such as hemodialysis, diabetes, cancer, frailty) [89,90,91,92,93,94,95,96,97,98]. Unlike a recognized high-risk population, IE may occur in a clinical context that is uncaptured by a conventional scoring approach. This group can be overlooked, challenging the ultimate diagnosis and treatment of IE. The untargeted at-risk population includes patients with a comorbidity burden determining recurrent bacterial exposure during healthcare access or intravenous catheter implantation. Recurrent highly virulent bacteremia, together with immune incompetence and endothelial inflammation due to comorbidity, favor the development of IE outside the expected conventional model. Varying prediction scores have been proposed to define a clinical expectation of IE and guide the subsequent imaging workflow. However, these emerging at-risk groups (especially patients with a comorbidity burden) are untargeted by risk score models, resulting in a potentially misleading workup. Consequently, a careful alert should be paved to identify patients who can benefit from a focused imaging workflow, despite a context of IE which is unexpected according to guideline recommendations. In the context of the untargeted at-risk population, an intriguing issue includes the differential diagnosis between infective valve vegetation and thrombotic/marantic noninfective valve masses [74,75,76,77,78,79,80]. Notably, cancer is associated with a high prevalence of thrombotic noninfective valve masses, together with a high occurrence of IE. Localization on the high-flow side, together with infiltrative characteristics and trajectory phenotype following antibiotic therapy, may suggest an infective etiology of valve masses. In selected patients with large valve masses, PET imaging may corroborate IE diagnosis, also providing a collateral finding of splenic uptake related to the infective state [27]. Heparin administration may be used in selected patients to enhance the differential diagnosis between thrombotic vs. infective masses. Finally, immunological conditions such as antiphospholipid syndrome or lupus may show valve masses, which require careful and skillful echocardiographic evaluation to confirm or rule out IE [78]. An intriguing clinical context of IE diagnostic criteria mismatch may be a consequence of the widespread use of imaging during opportunistic screening, including postcardiac surgery survey, cardiac follow-up, comorbidity survey, and noncardiac surgery workflow. The opportunistic imaging of typical valve lesions may suggest IE diagnosis, despite a clinical low-probability context. See Clinical Case 2.

Clinical Case 2. Unexpected endocarditis at opprtunistic echocardiography follow-up. A 60-year-old woman was observed at our heart valve clinic for a scheduled 3-month survey following surgical mitral valve repair of prolapse-related mitral regurgitation. The patient was asymptomatic under medical therapy, including a beta-blocking agent and routine early anticoagulation with dicumarol. Clinical examination was normal. Transthoracic echocardiography showed a small iso-echogenic mass on the atrial side of the posterior mitral leaflet. The subsequent transesophageal echocardiography (1 = 2D-TEE, 2 = 3D-TEE), showed a large iso-echogenic mass with an annular infiltrative appearance suggesting active valve vegetation. Blood cultures and phlogistic indices were normal. A PET examination (3 = PET) showed moderate mitral ring uptake, coherent with the recent surgical procedure. Due to the unexpected findings suggesting IE without clinical and microbiological associated criteria, TEE was re-evaluated following seven-day treatment with iv Heparin and empirical antibiotic therapy (Vancomicin, Gentamicin, Rifampicin). Owing to the persistence of mitral valve mass at high embolic risk, the patient underwent surgery. Surgical inspection (4 = surgical specimen) and histological examination confirmed IE diagnosis with a microbiological tissue culture for *Enterobacter Cloacae*. Following mitral valve replacement the clinical course was favorable with 1-month focused antibiotic therapy, without IE recurrence at long-term follow-up. This case underscores the importance of a careful and systematic survey following cardiac surgery to exclude silent unexpected endocarditis, especially during the first year after heart valve surgery.

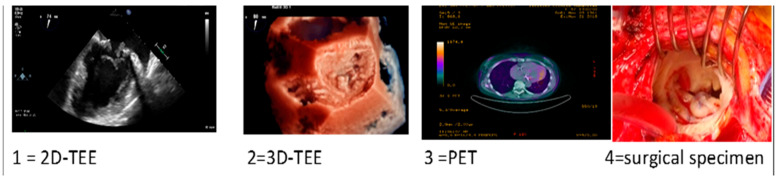


Healthcare-associated infections are a fearful risk for IE development in patients with a comorbidity burden (hemodialysis, diabetes, cancer, frailty) that are untargeted in conventional risk stratification. Frequent healthcare interventions, despite the absence of classic risk factors, can be at risk due to exposition to highly virulent nosocomial bacteria, leading to unexpected IE.

In these populations, IE can often be misdiagnosed late due to the absence of typical clinical features. Awareness and education regarding the potential risk for endocarditis in these less targeted groups are paramount for both healthcare providers and patients. Systematic check-ups and attention to symptoms such as unexplained fevers, heart murmurs, or persistent fatigue can facilitate early IE detection.

Healthy subjects without pre-existing at-risk cardiac disease, when exposed to recurrent highly virulent bacteremia, may also develop endothelial inflammation favoring IE in the absence of pre-existing valve damage. This subgroup includes skin-contact athletes, piercing implants drug addicts, the homeless, and alcoholics. In this subgroup, the mismatch between a clinically low-risk condition and typical IE features is an important source of unexpected diagnosis.

Finally, it is important to underline that varying healthcare systems and available resources may affect the generalizability of some of the above recommendations.

### 5.1. Clinical Phenotypes of Unexpected Infective Endocarditis Syndrome

Unexpected IE may occur in varying contexts, which can be clustered into clinical phenotypes that challenge the typical IE paradigm. A focused workflow should be triggered in the setting of these refined clinical contexts to enhance the timely diagnosis and treatment of IE.

#### 5.1.1. Hidden Subtle Clinical Phenotype

The hidden phenotype of IE is characterized by a subtle clinical context where the symptoms, signs, or clinical presentation are not typical or prominent, making accurate diagnosis challenging. This condition is a serious concern because atypical presentation leads to misinterpretation of the patient’s clinical conditions and can delay diagnosis and treatment, potentially resulting in severe and life-threatening complications. Typical IE symptoms, such as fever, a new or changed heart murmur, or signs of embolic phenomena, might be minimal or absent. On the contrary, hidden conditions may occur with subtle or atypical symptoms, including unexplained fatigue, as well as “loss of smile”, weight loss, low-grade fever, night sweats, or joint pain. Hidden IE is frequent in the elderly, immuno-compromised patients, hemodialysis patients, and those with a history of intravenous drugs or indwelling catheters. Patients might not show the risk factors typically associated with IE, such as pre-existing at-risk cardiac conditions. The hidden clinical phenotype highlights the importance of considering IE in the differential diagnosis of patients with persistent, unexplained symptoms, as well as in the absence of typical signs and risk factors. Early recognition and treatment are crucial to prevent serious complications, such as heart failure, systemic emboli, and the irreversible damage of heart valves. A high index of suspicion is required in patients with atypical symptoms or presentations. Detailed clinical evaluation and a history of cardiac disease, recent dental or medical procedures, or the presence of prosthetic heart valves may support the workup for IE. Prevention should promote awareness among healthcare professionals and the general public regarding this potentially life-threatening condition. Hidden IE underscores the importance of considering IE in the differential diagnosis of patients presenting with nonspecific symptoms, including those with relevant conventional risk factors or untargeted emergent at-risk conditions. See Clinical Case 3.

Clinical Case 3. Unexpected IE in at-risk patient. Female, 80 years old, who underwent previous breast cancer treatment (surgery, chemotherapy, radio therapy). Due to symptomatic severe mitral regurgitation, the patient underwent surgical mitral valve replacement with a biological prosthesis. A 3-month follow-up transthoracic echocardiogram (TTE) showed a normally functioning bioprosthesis. Six months after surgery, the patient was hospitalized for pulmonary edema coincident with high-ventricular rate atrial fibrillation responsive to medical treatment. During the subsequent month, the patient showed transient low-grade fever with a mild increase in PCR and leucocytes together with X-ray evidence of pulmonary infiltrate suggesting pneumonia. Thus, empirical antibiotic therapy was started in addition to cardiac medications (beta-blocking agent, angiotensin II inhibitor, edoxaban). A subsequent 3-day TTE (picture A) showed an unexpected iso-echogenic mass on the atrial surface of the mitral bioprosthesis. Due to concomitant anticoagulant therapy, IE was suspected instead of valve thrombosis with admission to our hospital. On admission the patient showed a stable clinical condition without fever or symptoms. Clinical examination showed a normal clinical condition with sinusal rhythm. Despite a low clinical probability of IE, due to echo-imaging, blood culture was carried out showing *S. epidermidis*. Focused antibiotic therapy, including Tazocin and Daptomicin, was started. Subsequent 6 h fasting Transesophageal Echocardiography (picture A = 2D-TEE; pictures 1–3 = 3D-TEE) showed a large iso-echogenic mass with bifurcated morphology (maximum diameter 25 × 10 mm). The mass was inserted on the atrial surface of the posterior prosthetic leaflet with annular infiltration and diastolic prolapse in the valvular ostium. Due to the high risk of embolism, the patient underwent a total body CT scan to exclude systemic embolization and subsequent emergency surgery. Surgical inspection (4, yellow arrow) and histological examination together with *S. epidermidis* isolation confirmed IE diagnosis. The clinical course was uneventful. This clinical case underscores the importance of clinical alert for unexpected at-risk IE requiring emergency surgery, despite a subtle and atypical clinical presentation.

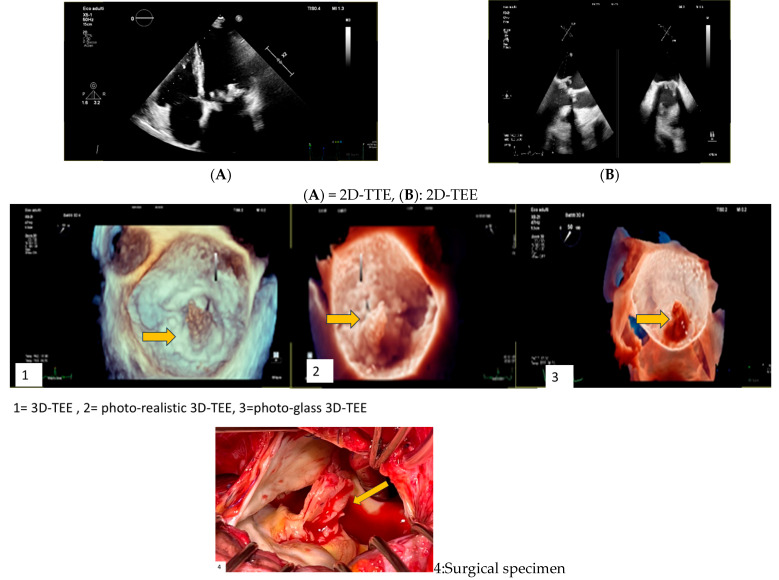


A subtle clinical phenotype of IE in elderly or frail patients deserves particular attention. Elderly patients have different risk factors, clinical presentations, and outcomes when compared with a younger population [82,83,84,85,86,87,88,89]. The classic clinical features of IE may be less prominent or even absent. The clinical picture may be characterized by nonspecific subtle symptoms, such as fatigue, confusion, or cognitive decline, making IE diagnosis challenging. Pre-existing cardiac risk conditions may be absent, and IE may overlap mild valve damage particularly caused by highly virulent bacterial healthcare-related contamination. Sometimes, frail elderly patients may show a dominant clinical feature of an explained isolated congestive heart failure before IE recognition [86]. The most common causative bacteria may include streptococcal species and healthcare-associated infections, such as *S. aureus* and enterococci. A high risk of complication may characterize the clinical presentation, with heart failure, systemic embolism, and stroke, which can open up the clinical scenario of IE. Diagnostic tests, such as blood culture, may be confounded by concurrent infection or catheter contamination and echocardiographic signs may be masked by redundant valve calcifications. The decision to operate is challenged by the presence of higher surgical risks and comorbidities. Preventive measures, such as maintaining good dental hygiene and the prompt treatment of any infections, are crucial. Multidisciplinary care is required. Early recognition, the prompt initiation of appropriate antibiotic therapy, and careful monitoring for complications are crucial in the management of IE in elderly and frail patients. Owing to these complexities, a tailored approach to a patient’s overall health status, comorbidities, and personal preferences is essential.

#### 5.1.2. Long-Term Extracardiac Symptoms

Varying long-term extracardiac features may subtly underly IE before the diagnosis is definitively confirmed. These symptoms can be consequent to infection, immune response, or embolic events [5,6]. In a clinically subtle context, some extracardiac symptoms may be prominent, including joint and muscle pain (arthralgias and myalgias) due to immune complex deposition or the phlogistic response, neurological changes as a consequence of embolic events, skin manifestation [97], such as subungual hemorrhages, Osler nodes (painful nodes on fingers or toes) and Janeway lesions (painless lesions on palms or soles), splenic enlargement or pain due to embolic phenomena, ocular changes [98], such as Roth spot (retinal hemorrhages with pale center), weight loss and night sweats, and hematuria or renal dysfunction as a consequence of immune complex deposition. These symptoms are not specific and can be mistaken for other conditions that challenge initial IE diagnosis [5,6,97,98].

#### 5.1.3. Infective Endocarditis with Dominant Spondylodiscitis

Spondylodiscitis is a subtle clinical manifestation of IE, occurring through bloodstream dissemination of bacteria responsible for cardiac infection, involving the spine and, more specifically, the intervertebral disks and the adjacent vertebral bodies [99,100,101]. The infection can lead to severe pain and other neurological symptoms due to nerve and spinal cord compression or involvement. Spondylodiscitis can be a dominant feature and may precede clinically overt IE development or diagnosis. The clinical scenario may be complex when the patient presents symptoms that are more indicative of spinal infection and/or compression, such as severe back pain, potentially overshadowing the typical signs and delaying a diagnosis of IE. Diagnosis is typically carried out with imaging tests such as MRI or CT and PET, as well as blood tests to identify the spinal infection. The identification of the same pathogen in both spinal and cardiac lesions corroborates a strong relationship between the two infections. The scenario is more common in older adults with pre-existing spine conditions or in immunocompromised individuals. In the subtle context, spondylodiscitis may be the first clinical feature of IE with prominent severe back pain, stiffness, and neurological deficit if the spinal cord or nerves are affected. It is important for individuals with spondylodiscitis, also in the absence of conventional heart disease risk, to carefully consider IE as a potential cause of spine infection, especially in the elderly or immunocompromised patients. A patient may at onset experience nonspecific symptoms (fatigue, mild or absent fever, joint pain), which can easily be mistaken for other diseases. In this condition, IE may be challenging and may be initially unsuspected, especially in the absence of heart-related symptoms or predisposing at-risk cardiac conditions. This scenario underscores the importance of a comprehensive medical evaluation when a patient presents with spondylodiscitis [102]. Careful investigation for a primary source of infection, including IE, is crucial. It requires a high degree of clinical suspicion and a multidisciplinary approach to its treatment and management. Close monitoring for response to treatment and potential complications is crucial, including repeated imaging and laboratory tests focusing on the effectiveness of therapy and monitoring for complications. See Clinical Case 4.

Clinical Case 4. Unexpected clinically silent IE recurrence. A 76-year-old man undergoing previous Bentall aortic surgery with a bioprosthetic valve. The patient remained asymptomatic for 6 years after surgery until the appearance of lumbar pain (subsequent diagnosis of spondylodiscitis at magnetic resonance imaging) without fever and Reactive C Protein increase. Due to *Streptococcus Bovis* bacteremia, the patient underwent TTE and TEE, showing mobile vegetation attached to the ventricular face of the aortic prosthetic leaflet. Following specific antibiotic therapy, TEE (**1A**) and CT scan (**1B**) showed a successful outcome with vegetation disappearance in the absence of clinical events related to a systemic embolization. In the absence of symptoms, to assess spondylodiscitis status, the patient underwent PET (**2A**–**C**), showing complete recovery from the spinal infection with concomitant imaging of abnormal FDGuptake involving the posterior side of the ascending aortic prosthesis and valvular prosthetic ring, suggesting a recurrence of bioprosthesis endocarditis with paraprosthetic ascending aortic abscess (red arrow). The findings were confirmed with subsequent CT (**2D**), TEE (**2E,F**) (yellow arrow), and surgical specimen (3). Blood cultures and histopathological examination were positive for *Candida Albicans* 
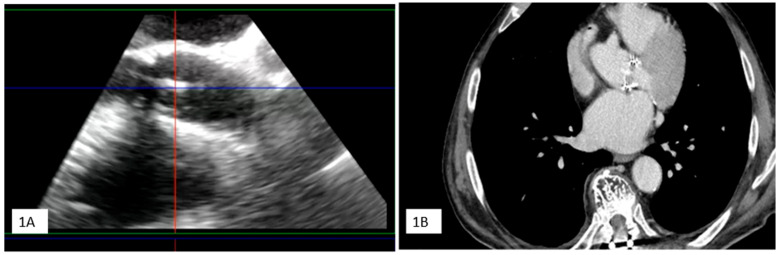
 
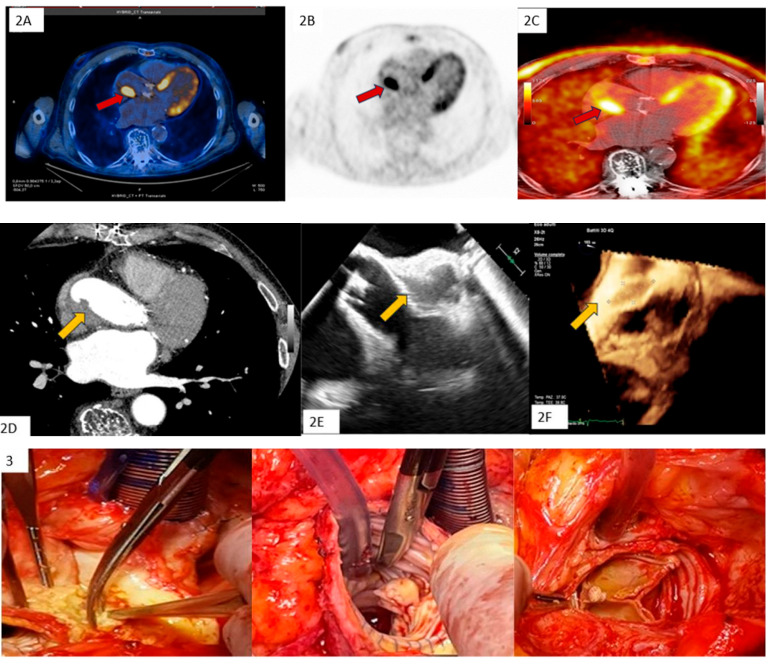

#### 5.1.4. Healthy Subjects

Healthy subjects may develop IE due to highly virulent bacteria, despite the absence of pre-existing at-risk cardiac conditions [103]. The clinical picture is dominated by classic symptoms, but sometimes extracardiac embolism may open a clinical IE scenario leading to a misleading diagnostic work-up [104].

#### 5.1.5. Deceptive Acute Clinical Context

Endocarditis may occur in an acute deceptive clinical context, where endocardial infection may occur in the setting of acute, also sudden and severe, medical conditions without a clinical alert of cardiac infection [105,106,107,108,109,110,111]. In this context, IE clinical presentation may be misleading, or not straightforward, complicating its diagnosis and management. The usual symptoms of IE, such as fever, heart murmur, or evidence of a new or changing heart valve lesion, might be absent or overshadowed by concomitant clinical features [108,109]. Misleading presentation commonly occurs with symptoms mimicking acute extracardiac conditions, such as respiratory or gastrointestinal infection, stroke, rheumatologic diseases, or localized infections, which can avert a consideration of endocarditis. In addition, IE may overlap in the context of an acute extracardiac pathology requiring intensive care stay. Due to these challenges, IE in an acute context is a serious medical concern requiring a high level of clinical vigilance for emergent risk conditions to look beyond the obvious or most common diagnoses [111].

The recognition of IE in a critical care setting requires careful clinical assessment and awareness when it may vary with fever, heart murmurs, signs of embolic events, or symptoms related to complications such as heart failure or stroke. A medical history regarding pre-existing at-risk heart conditions, a history of intravenous drug use, recent dental or surgical procedures, or the presence of prosthetic heart valves or indwelling catheters is important. Blood cultures are essential to identify the causative organism. Multiple cultures may be needed over time to increase the chances of detection, especially in patients already undergoing antibiotic treatment. Echocardiography using the transesophageal approach is crucial to carry out diagnosis and detect complications. Additional CT scans or MRIs may be required to identify complications, such as embolic events, or to assess the extent of the infection. The application of diagnostic Duke criteria may be misleading due to confounding conditions. The consideration of a differential diagnosis in a deceptive acute context is essential to evaluate other diagnoses that can mimic IE, such as thrombotic thrombocytopenic purpuras, disseminated intravascular coagulation, or other types of sepsis. Consultation with multiple specialists is crucial. In a critical care context, the prompt and accurate diagnosis of IE is essential to guide appropriate and potentially life-saving treatment. The process involves a combination of clinical assessment, laboratory testing, imaging, and collaborative decision making. In some deceptive cases, blood culture may be a false negative due to prior antibiotic administration or because some organisms are difficult to culture. Additional false-positive cultures may occur due to contamination or extracardiac foci. See Clinical Case 5.

Clinical Case 5. *Streptococcus pneumoniae* infection with meningitis, pneumonia, and fulminant endocarditis (“Austrian syndrome”). A 72-year-old female was admitted after 3 days of fever, shortness of breath, and abrupt headache with nausea and vomiting. On admission, the patient appeared confused and troubled. Clinical examination revealed nuchal rigidity without focal neurologic signs. Her temperature was 39 °C, arterial blood pressure 115/60 mm Hg. The clinical diagnosis was initially addressed toward bacterial or viral meningitis vs. COVID-19 with meningoencephalitis phenotype. Lumbar puncture was carried out; cerebrospinal fluid analysis showed hypoglycorrhachia and hyperproteinorrhachia, supporting a diagnosis of *probable bacterial meningitis*. Empirical antibiotic therapy with intravenous Ceftriaxone, Vancomycin and Dexametasone, was promptly started. A brain CT was negative for intracranial lesions. Baseline and contrast CT showed normal pulmonary findings without parenchymal consolidation or signs of thromboembolism. Pneumococcal antigen in urine was positive and blood culture (BC) showed Gram-positive cocci. Nasopharyngeal Swab (NS) was negative for COVID-19. The final BC result confirms *S. pneuomoniae* bacteremia, supporting a bacterial meningitis etiology. Targeted antibiotic therapy was introduced, including levofloxacin. After 12 h, the patient showed abrupt cognitive impairment with arterial blood hypotension (80/50 mm Hg) and severe hypoxemia requiring endotracheal intubation. The patient was admitted to the intensive care unit with a diagnosis of pneumococcal meningitis and septic shock. After two days, due to significant clinical improvement, endotracheal intubation was removed. However, during the subsequent two hours the patient showed atrial fibrillation with a high ventricular rate that was reverted with iv amiodarone. Clinical examination revealed a new diastolic regurgitant murmur at cardiac auscultation. Temperature was 37.5°, blood pressure 155/50 mm Hg. Large vegetation with severe AR was shown by TTE. Subsequent TEE confirmed AR due to extensive damage of three valve leaflets with large iso-echogenic (like myocardium echogenicity) vegetation, inserted at the level of the ventricular surface of the right coronary cusps (dimension 20 × 7 mm) and prolapsing in the left ventricular outflow tract. There was moderate MR, pulmonary artery hypertension (sPAP 70 mm Hg), and severe TR. Left ventricular dimension and ejection fraction were normal. A fresh thrombus was found in the left atrial appendage. Inotropic support, with furosemide and heparin infusion, was started. Due to high systemic embolic risk (large aortic valve vegetations and left atrial appendage thrombosis), total body CT was performed. Brain CT was normal, while the thoracic scan revealed bilateral parenchymal consolidation and large pleural effusion with parenchymal edema (Figure 1). Additional NPS and BAL excluded COVID-19. Final diagnosis was “*Austrian syndrome*” (6), including meningitis, with subsequent pneumoniae and endocarditis due to *S. pneumoniae*. Although clinically indicated, cardiac surgery was postponed due to the unavailability of an operating room during the COVID-19 outbreak. The next day, hemodynamics remained stable under inotropic, diuretic, and mechanical ventilation support. A new TEE showed unchanged findings, apart from the disappearance of the thrombus in the left atrial appendage. The following day, the patient underwent contrast CT, showing significant improvement of pulmonary findings, a normal coronary anatomy, and double bulging of the aortic root below the coronary artery ostium. Immediate TEE was performed showing, in addition to the previous findings, an intimal tear with a new cavity of the anterior wall of the aortic root at the site of systolic contact of vegetation, suggesting a “kissing” mycotic pseudoaneurysm. In addition, TEE revealed normalization of sPAP and related TR. The patient, due to the added risk of impending root rupture, underwent emergency cardiac surgery. Anatomical inspection confirmed large vegetation and extensive leaflet damage of the tricuspid aortic valve, with a large paravalvular mycotic pseudoaneurysm (Figure 1). The operating strategy involved drainage of purulent material and pseudoaneurysm patch repair, followed by biological valve prosthesis implantation. The leaflet aortic culture was positive for *S. Pneumoniae*. The postoperative clinical course was uneventful, with complete recovery of the clinical condition. Comment. This case outlines the importance of careful daily clinical examination to target the appropriate pathway for the identification and management of unexpected fulminant IE, which can be superimposed in the deceptive context of meningitis and pneumonia due to *S. pneumoniae* infection (so-called Austrian syndrome).

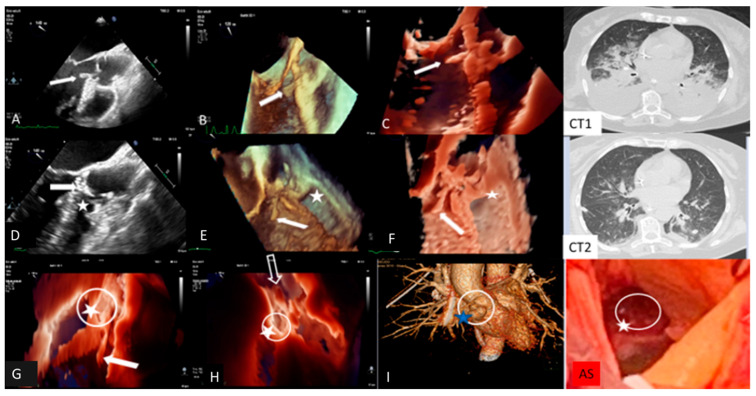
Transesophageal echocardiography and computed tomography of fulminant aortic endocarditis. TEE images: (**A**) two-dimensional; (**B**) three-dimensional; (**C**): photorealistic three-dimensional showing large vegetation (arrow) on the aortic valve prolapsing during diastole into LVOT, increasing at 24 h TEE images (**D**–**F**) together with an intimal tear communicating with a neocavity (star) on the aortic anterior wall at the site of vegetation contact (dotted arrow), suggesting a mycotic pseudoaneurysm; (**I**): three-dimensional computed tomography showing double bulging of the anterior aortic wall (dotted circle) overlapping photorealistic 3D images (**G**,**H**) and surgical specimen findings (**AS**). Computed tomography showing bilateral pulmonary parenchymal consolidation and large pleural effusion (**CT1**), which improved following thoracentesis and intensive medical therapy (**CT2**).

## 6. Conclusions

The conventional paradigm of IE may be subverted towards an unexpected clinical scenario, occurring with atypical clinical features, or involving subjects without pre-existing at-risk cardiac disease. Owing to its unexpected clinical context, IE might be overlooked, resulting in a challenging diagnosis and delayed treatment. Unexpected IE should be regarded as a complex syndrome, subtending a varying clinical spectrum with subtle and deceptive phenotypes. The awareness of an unexpected clinical course should alert clinicians to also consider IE diagnosis in patients with atypical features, enhancing vigilance for preventive measures in an emergent untargeted at-risk population.

## Supplementary Materials

The following are available online at https://www.mdpi.com/article/10.3390/jcm13175058/s1, Table S1, Clinical score models intended to assess expected IE risk deserving subsequent transesophageal echocardiography assessment.
