# Supplementary material for: Unexpected Infective Endocarditis: Towards a New Alert for Clinicians
Source: J Clin Med. 2024 Aug 26;13(17):5058. doi: 10.3390/jcm13175058 (PMC11396651; doi:10.3390/jcm13175058)
Supplement: Supplementary file 1 [file jcm-13-05058-s001.zip › jcm-3108640-supplementary.pdf]

Table S1. Clinical score models intended to assess expected IE risk deserving subsequent transesophageal echocardiography assessment.

Study	Bacteremia	Trial	Score Variables	Risk score Threshold for TEE	Diagnostic power	Limitations	
**PREDICT**<br>Mayo Clinic<br>Rochester	***Staphylococcus aureus***	Retrospective<br>678 pts	Day 1 Screening:<br>1. Onset of SAB: community acquired (2), hospital care-associated (1), nosocomial (0)<br>2. Presence of CIED: implantable cardioverter defibrillator (2), permanent pacemaker (3)<br>Day 5 Screening:<br>1. Community onset SAB (2)<br>2. Presence of CIED, implantable cardioverter defibrillator (2), permanent pacemaker (3)<br>3. Prolonged bacteremia-persistently positive blood cultures for ≥72 hours (2)	-score ≥4 on day 1 screening<br>-score ≥2 on day 5 screening	Sensitivity 51.6%/NPV 95.1%<br>Sensitivity 85.1%/NPV 94.5%<br>Sensitivity Day 1 score ≥4: 30.4%<br>Specificity Day 1 score ≥4: 93.8%<br>Specificity Day 5 score ≥2: 41.1%<br>Sensitivity Day 5 score ≥2: 100%/NPV 100%	Based on SAB characteristic<br>Predisposing factors limited to CIED or intracardiac catheters presence	
**VIRSTA**<br>8 tertiary-care University French hospitals	***Staphylococcus aureus***	Prospective cohort Study<br>2008 pts	1. Meningitis (5)<br>2. Cerebral or extracerebral emboli (5)<br>3. Intracardiac device or previous IE (4)<br>4. IVDU (4)<br>5. Pre-existing native valve disease (3)<br>6. Persistent bacteremia for ≥48 hours (3)	day 1 and 3 of SAB diagnosis calculated score ≥3	Sensitivity 96.7%/NPV 99.5%<br>Sensitivity 98.9%/NPV 99.3%<br>Sensitivity: 94.4%	Low number of patients who underwent TEE in the study<br>Most patients were recruited from tertiary care centers<br>Needs external validation	

			7. Community or non-nosocomial acquisition (2)<br>8. Vertebral osteomyelitis (2)<br>9. Severe sepsis or septic shock (1)<br>10. C-reactive protein >190 mg/L (1)			of finding from other countries	
**POSITIVE**<br>10 hospitals in Sweden	***Staphylococcus aureus***	Retrospective population-based study<br>465 episodes	1. Time to positivity (TTP):<br>TTP < 9 hr (5 pts)<br>TTP ≥9 hr but <11 hr (3 pts)<br>TTP ≥11 hr but <13 hr (2 pts)<br>2. IV drug use (3 pts)<br>3. Vascular phenomena (6 pts)<br>4. Predisposing heart disease (5 pts)	A score >4 indicative of high risk for IE and needs TEE	Sensitivity 77.6%/NPV 92.5%<br>Sensitivity 77.8%	1. Retrospective study unable to control different lab collection variables that can impact TTP<br>2. Low number of patients who underwent TEE in the study	
**HANDOC**<br>Database of the Laboratory for Clinical Microbiology in Lund,<br>Sweden	**Non-β-Hemolytic *Streptococcus***	Retrospective cohort study<br>339 patients	1. Heart murmur or valve disease (1 pt)<br>2. Etiology with the groups of *S. mutans, S. bovis, S. sanguinis, or S. anginosus* (1 pt)<br>3. Number of positive blood cultures ≥2 (1 pt)<br>4. Duration of symptoms of ≥7 days (1 pt)<br>5. Only 1 species growing in blood cultures (1 pt)<br>6. Community acquired infection (1 pt)	A score ≥3 indicative of higher risk for IE and needs TEE.	Sensitivity 100%<br>Specificity 76%	1. Retrospective study<br>2. The group of patients with IE was too small to allow multivariable analysis.<br>3. Excluded patients with neutropenia	
**NOVA**<br>1550-bed tertiary center in Spai	***Enterococcus***	Prospective cohort study and case control study<br>1515 patients	1. Number of positive blood cultures suggestive of continuous bacteremia (5 pts)<br>2. Unknown origin of bacteremia (4 pts)	A score >4 points indicative of higher risk for IE and needs TEE.	Sensitivity 97%/NPV 95%<br>Specificity 23%/PPV 38%	1. Single-center prospective cohort study<br>2. Selection bias from the intervention of infectious disease specialist	

			3. Prior valve disease (2 pts)<br>4. Heart murmur (1 pt)				
**DENOVA**<br>Database of the Laboratory for Clinical Microbiology in Skåne County, in Sweden.	***Enterococcus***	Retrospective population based cohort study	1. Duration of symptoms ≥7 days (1 pt)<br>2. Embolization (1 pt)<br>3. Number of positive cultures ≥2 (1 pt)<br>4. Origin of infection unknown (1 pt)<br>5. Valve disease (1 pt)<br>6. Auscultation of murmur (1 pt)	A score ≥3 indicative of higher risk for IE and needs TEE.	Sensitivity 100%<br>Specificity 85%<br>Validation cohort was from Karolinska University Hospital, a tertiary referral center Serving Stockholm	1. Retrospective study with concerns for misclassification bias<br>2. Score cannot be applied to *E. faecium* bacteremia and polymicrobial *E. faecalis* bacteremia<br>3. Low number of patients who underwent TEE in the study	
